# Supplementary material for: Mutations in PCYT2 disrupt etherlipid biosynthesis and cause a complex hereditary spastic paraplegia
Source: Brain. 2019 Oct 22;142(11):3382–97. doi: 10.1093/brain/awz291 (PMC6821184; doi:10.1093/brain/awz291)
Supplement: awz291_Supplementary_Data [file awz291_supplementary_data.zip › awz291-Suppl_data/Supplementary_Data4.zip!/Supplementary information 4 - plasma lipidomics.zip!/Plasma lipidomics/Control vs Patient1/Statistical plots and data/Random forest.pdf]

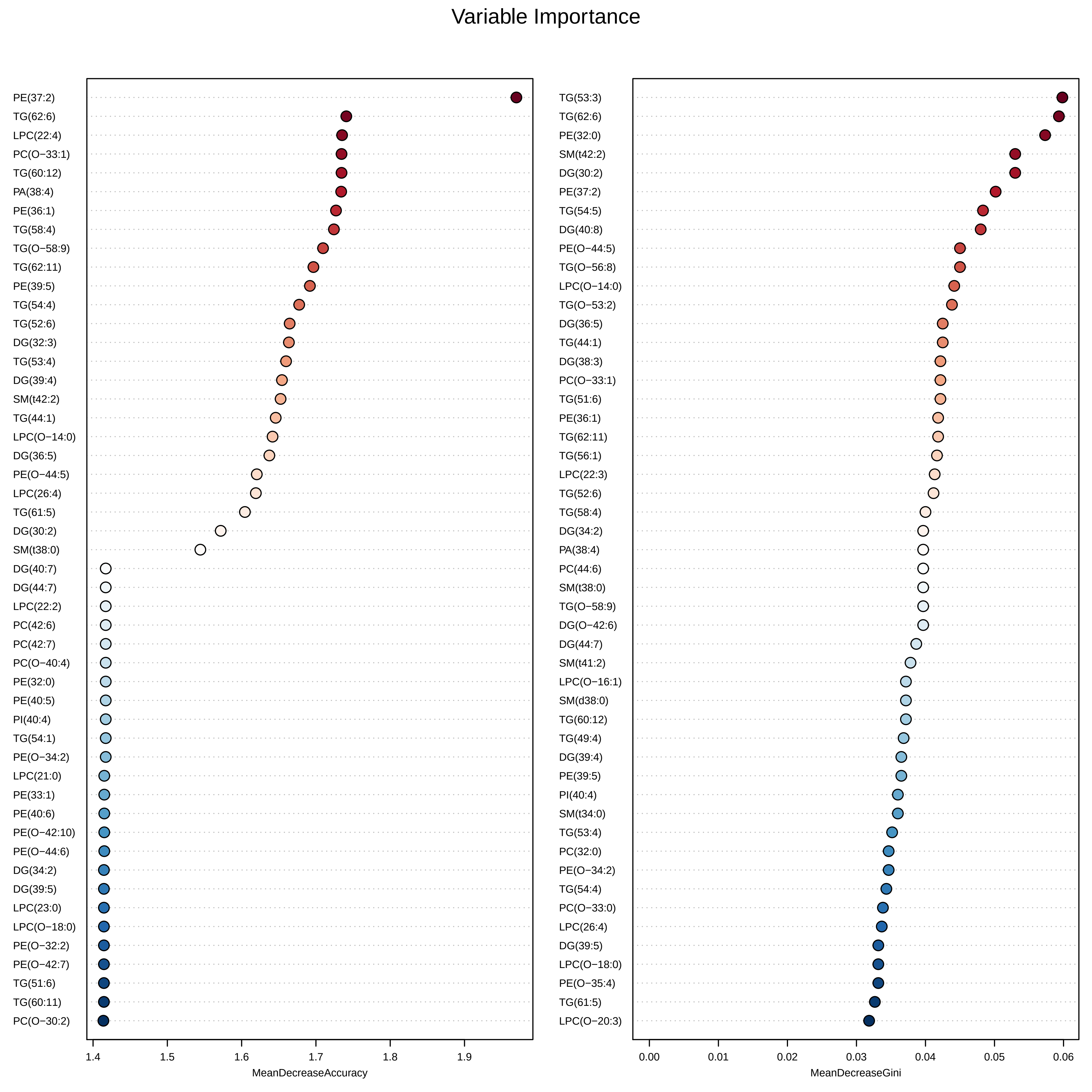
Variable Importance
PE(37:2)
TG(62:6)
LPC(22:4)
PC(O−33:1)
TG(60:12)
PA(38:4)
PE(36:1)
TG(58:4)
TG(O−58:9)
TG(62:11)
PE(39:5)
TG(54:4)
TG(52:6)
DG(32:3)
TG(53:4)
DG(39:4)
SM(t42:2)
TG(44:1)
LPC(O−14:0)
DG(36:5)
PE(O−44:5)
LPC(26:4)
TG(61:5)
DG(30:2)
SM(t38:0)
DG(40:7)
DG(44:7)
LPC(22:2)
PC(42:6)
PC(42:7)
PC(O−40:4)
PE(32:0)
PE(40:5)
PI(40:4)
TG(54:1)
PE(O−34:2)
LPC(21:0)
PE(33:1)
PE(40:6)
PE(O−42:10)
PE(O−44:6)
DG(34:2)
DG(39:5)
LPC(23:0)
LPC(O−18:0)
PE(O−32:2)
PE(O−42:7)
TG(51:6)
TG(60:11)
PC(O−30:2)
1.4
1.5
1.6
1.7
1.8
1.9
MeanDecreaseAccuracy
TG(53:3)
TG(62:6)
PE(32:0)
SM(t42:2)
DG(30:2)
PE(37:2)
TG(54:5)
DG(40:8)
PE(O−44:5)
TG(O−56:8)
LPC(O−14:0)
TG(O−53:2)
DG(36:5)
TG(44:1)
DG(38:3)
PC(O−33:1)
TG(51:6)
PE(36:1)
TG(62:11)
TG(56:1)
LPC(22:3)
TG(52:6)
TG(58:4)
DG(34:2)
PA(38:4)
PC(44:6)
SM(t38:0)
TG(O−58:9)
DG(O−42:6)
DG(44:7)
SM(t41:2)
LPC(O−16:1)
SM(d38:0)
TG(60:12)
TG(49:4)
DG(39:4)
PE(39:5)
PI(40:4)
SM(t34:0)
TG(53:4)
PC(32:0)
PE(O−34:2)
TG(54:4)
PC(O−33:0)
LPC(26:4)
DG(39:5)
LPC(O−18:0)
PE(O−35:4)
TG(61:5)
LPC(O−20:3)
0.00
0.01
0.02
0.03
0.04
0.05
0.06
MeanDecreaseGini
